# Sequencing the cap-snatching repertoire of H1N1 influenza provides insight into the mechanism of viral transcription initiation

**DOI:** 10.1093/nar/gkv333

**Published:** 2015-04-21

**Authors:** David Koppstein, Joseph Ashour, David P. Bartel

**Affiliations:** 1 Department of Biology, Massachusetts Institute of Technology, Cambridge, MA 02139, USA; 2 Whitehead Institute of Biomedical Research, 9 Cambridge Center, Cambridge, MA 02142, USA; 3 Howard Hughes Medical Institute, Whitehead Institute of Biomedical Research, Cambridge, MA 02142, USA

## Abstract

The influenza polymerase cleaves host RNAs ∼10–13 nucleotides downstream of their 5′ ends and uses this capped fragment to prime viral mRNA synthesis. To better understand this process of cap snatching, we used high-throughput sequencing to determine the 5′ ends of A/WSN/33 (H1N1) influenza mRNAs. The sequences provided clear evidence for nascent-chain realignment during transcription initiation and revealed a strong influence of the viral template on the frequency of realignment. After accounting for the extra nucleotides inserted through realignment, analysis of the capped fragments indicated that the different viral mRNAs were each prepended with a common set of sequences and that the polymerase often cleaved host RNAs after a purine and often primed transcription on a single base pair to either the terminal or penultimate residue of the viral template. We also developed a bioinformatic approach to identify the targeted host transcripts despite limited information content within snatched fragments and found that small nuclear RNAs and small nucleolar RNAs contributed the most abundant capped leaders. These results provide insight into the mechanism of viral transcription initiation and reveal the diversity of the cap-snatched repertoire, showing that noncoding transcripts as well as mRNAs are used to make influenza mRNAs.

## INTRODUCTION

In eukaryotic gene expression, a 7-methylguanosine (m^7^G) cap is added to the beginning of an mRNA by a 5′-5′ triphosphate and is important for stability, export, and translation of that transcript (1–3). This cap dependence poses a challenge for RNA viruses because many are unable to use the cellular RNA capping machinery, which is associated with DNA-dependent RNA polymerase II (Pol II) (4,5). These viruses have developed diverse strategies to circumvent this problem. Some encode their own capping machinery, some covalently attach a viral protein to the mRNA 5′ terminus, others use internal ribosome entry sites, and several negative-stranded, segmented ssRNA viruses use an unusual strategy termed ‘cap-snatching’ to steal short 5′ fragments of cellular mRNAs and use these capped fragments for the synthesis of viral mRNAs (6).

Cap-snatching was first discovered in the influenza virus (7,8), which remains the best-characterized system for this phenomenon. The influenza RNA-dependent RNA polymerase (RdRP), comprised of the PA, PB1 and PB2 proteins, is recruited to promoter-associated Pol II and cleaves cellular mRNAs ∼10–13 nucleotides from their 5′ end and uses the resulting cleavage product to prime viral mRNA transcription (9,10). PB2 binds the cap of the cellular mRNAs (11–13), PA is responsible for the endonuclease cleavage (14,15), and PB1 contains the polymerase activity (16,17). The polymerase is also responsible for polyadenylation of viral mRNAs and replication of the negative-stranded viral genome (vRNA), which consists of eight RNA segments, through complementary RNA (cRNA) intermediates (6,18–20).

Because early experiments on influenza cap-snatching were performed in rabbit reticulocyte lysate, where α- and β-globin mRNAs are highly expressed, these transcripts were the first cap-snatched substrates identified (7–8,21). Studies on influenza cap-snatching in different cell types revealed that the host leaders prepended to viral mRNAs have highly heterogeneous sequences, indicating that influenza polymerase targets diverse mRNAs (9–10,22–25). Two early studies reported that these host-derived heterogeneous sequences often end with a CA or GCA, suggesting that a specific subset of mRNAs are targeted by the cap-snatching machinery (10,25) and leading to the idea that a preference for certain messages might be important for viral fitness, perhaps by suppressing the expression of antiviral factors (26).

Several lines of evidence have been put forward to suggest that the influenza polymerase exhibits sequence specificity. A preference for CA dinucleotides at the ends of host-derived heterogeneous sequences is suggested to arise from sequence specificity of the viral endonuclease and preferential priming of CA-terminated RNA fragments (27). Subsequent analysis shows that the N-terminal domain of PA endonuclease does indeed have sequence selectivity *in vitro*, but that it preferentially cleaves after G residues (26). Results of other experiments are interpreted to indicate that an AGC consensus sequence at the 3′ termini of host-derived sequences might base pair to the viral template to promote efficient transcription initiation (28–30), in contrast with an earlier model which postulated priming without base pairing (31). Although these findings and their mechanistic interpretations are not fully consistent with each other, together they suggest some intrinsic specificity of the polymerase, possibly mediated by PA endonuclease, base pairing to the viral template, or some combination thereof.

Another mechanism that could explain the sequence preferences reported at the ends of heterogeneous sequences is polymerase slippage during transcription initiation. Viral polymerase slippage could lead to reiterative copying of the 3′ end of influenza vRNAs, potentially explaining the enrichment of CA and GCA at the heterogeneous-sequence 3′ termini (25). This mechanism was revisited when repeats matching the viral template were found in hantavirus mRNAs, which are also primed by cap-snatched host fragments. Further analysis of the slippage phenomenon led to the ‘prime-and-realign’ model, in which the nascent transcript sometimes shifts back several nucleotides to reiterate transcription of some of the first template residues (32). Nucleotide patterns consistent with prime-and-realign have since been found in several related clades of viruses that snatch-capped leaders from host mRNAs, including the *Bunyaviridae* and *Arenaviridae* families, as well as their plant-infecting, negative-stranded counterparts, the Tenuiviruses (33–40). Importantly, recent studies of influenza cap-snatching that employed a defined mRNA substrate reported untemplated nucleotides at the junction between the capped substrate fragment and the viral mRNA, indicating that prime-and-realign can occur for influenza as well (28,29). The ability of the influenza virus polymerase to slip in certain contexts is also consistent with the mechanism of viral mRNA polyadenylation, during which the viral polymerase stutters over a six-nucleotide U track (18,19).

To evaluate these mechanistic possibilities, we used high-throughput sequencing to profile the 5′ ends of A/WSN/33 influenza mRNAs during infection of human lung epithelial cells, thereby providing a global cap-snatching repertoire of H1N1 influenza *in vivo*. In the meantime, another study has also used similar methods to investigate the *in vivo* cap-snatching repertoire of H3N2 influenza (41). The authors of that study find that different viral transcripts have striking differences in host-derived heterogeneous sequences and interpret this as a surprisingly divergent specificity in the cellular transcripts that contribute leaders to the different viral mRNAs. In contrast, we systematically distinguished the contribution of prime-and-realign from the intrinsic cleavage and priming specificities of the influenza RdRP and reached the opposite conclusion, in which essentially indistinguishable sets of cellular transcripts contribute leaders to the different viral mRNAs. Moreover, the identity of the host transcripts revealed that not all the leaders derive from host mRNAs; many are from host noncoding RNAs, including small nuclear RNAs (snRNAs) and small nucleolar RNAs (snoRNAs). Our data also support a mechanism of viral transcription initiation in which a single Watson–Crick (or G:U wobble) base pair is sufficient to prime viral transcription.

## MATERIALS AND METHODS

### Virus propagation and infection

A549 cells (ATCC) were propagated in Dulbecco's Modified Eagle Medium (Gibco) supplemented with 10% fetal bovine serum (FBS) and pen/strep (100 U/ml penicillin, and 100 mg/l streptomycin, Cellgro). A day before infection, confluent cells in a T-75 flask were diluted 4-fold and split into six-well plates. Three wells were washed twice with 2 ml of phosphate-buffered saline (PBS) supplemented with Ca^2+^ and Mg^2+^ (PBS+). Five hundred microliters infection media, consisting of PBS+ w/ 0.2% bovine serum albumin (BSA), pen/strep and A/WSN/33 (H1N1) influenza at a final MOI of 1–3, was added to each well after vortexing briefly. RNA from three wells was harvested with TRI reagent (Life Technologies) according to protocol after the indicated duration of infection (Supplemental Table S1).

### High-throughput sequencing of influenza mRNA 5′-termini

Five to ten micrograms RNA was diluted in 50 mM NaCl and 3.3 μM gene-specific outer primer (Supplemental Table S2) and adjusted to a volume of 6 μl. The mixture was heated to 65°C for 5 min, followed by a cooling ramp (0.1°C/s) to 4°C. In parallel, 2 μl of 100 μM template-switching oligonucleotide (TSO, IDT, Supplemental Table S2) was denatured at 65°C for 5 min and snap-cooled on ice. These were combined in a SuperScript II mixture (Life Technologies) with sorbitol/trehalose and betaine as described previously (42). After 60 min at 42°C, RNA was hydrolyzed by adding NaOH to a final concentration of 130 mM at 90°C for 10 min, then quenched with 5-fold molar excess of prechilled 1 M HEPES pH 7, extracted with phenol:chloroform:isoamyl alcohol 25:24:1 pH 8 (Sigma–Aldrich) and chloroform. cDNA was precipitated with Pellet Paint NF co-precipitant (EMD Millipore) in ethanol at −20°C, resuspended in 1× Gel Loading Buffer II (Life Technologies) and gel-purified on a thin denaturing 6% polyacrylamide gel, excising the slice corresponding 200–1000 nt, eluting in 800 μl of 0.3 M NaCl at room temperature overnight, and precipitating as before. cDNA was then PCR amplified with one common primer and one gene-specific primer (Supplemental Table S2), using Phusion HF according to the manufacturer's protocol (New England Biolabs) with an extension time of 30 s, for 21–27 cycles. The resulting PCR product was precipitated and gel-purified as before, this time excising the corresponding PCR product by SYBR gold staining (Life Technologies). Following precipitation, libraries were resuspended in 10 μl Tris pH 8.0, and sequenced on an Illumina HiSeq 2000.

### Data analysis

Computational analysis was performed on an LSF cluster using custom Python scripts (43–47). A more complete description of the algorithms used is detailed in the supplemental materials. The full pipeline is implemented as a Makefile, and all source code is available on the Bartel Lab website (http://bartellab.wi.mit.edu/publication.html). Raw data are available from the NCBI GEO database, accession number GSE65101.

## RESULTS

### High-throughput sequencing of the 5′ ends of influenza mRNAs

**Figure 1.**
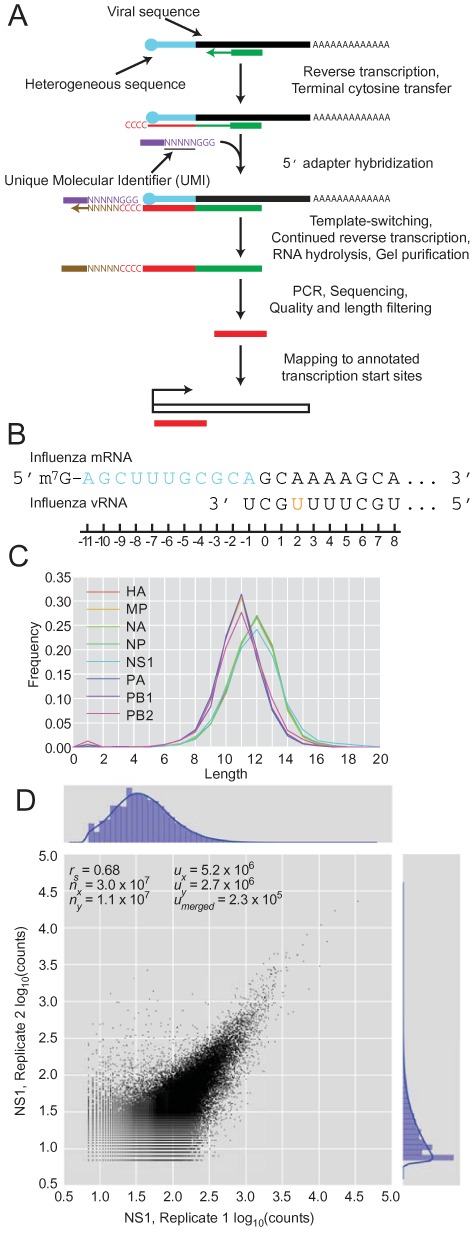
High-throughput sequencing of the heterogeneous 5′ ends of influenza mRNAs. (**A**) Schematic of the sequencing method and subsequent mapping. (**B**) Diagram of the relevant regions of the influenza mRNA and vRNA template. The heterogeneous sequence is in blue, and the remainder of the mRNA is in black. An orange nucleotide denotes the SNP in the 3′ region of vRNA that differs between viral genes. (**C**) Length distributions of the heterogeneous sequences grouped by influenza mRNA. (**D**) Number of reads corresponding to the same heterogeneous sequences in two biological replicates for the NS1 gene at 4 h.p.i. *r*_s_, Spearman *r* coefficient; *n*_x_ and *n*_y_, number of reads in each dataset; *u*_x_ and *u*_y_, number of unique sequences in each dataset; *u*_merged_, number of unique sequences in the intersection of the compared datasets.

To profile the cap-snatching repertoire of influenza, we modified a high-throughput 5′-RACE (rapid amplification of cDNA ends) method previously used in single-cell transcriptomics (48) (Figure 1A). The approach utilizes the template-switching activity of reverse transcriptase to install an adapter at the 3′ end of the cDNA (5′ end of the viral mRNA), which includes a random barcode, or unique molecular identifier (UMI), that is useful for identifying uniquely sequenced ends (49–52). We modified this method by using reverse-transcription primers that hybridized specifically to each of the eight viral mRNAs, which generated datasets for each of the eight influenza mRNAs (and a biological replicate for the NS1 mRNA), containing five hundred thousand to 31 million reads per dataset after collapsing PCR duplicates (Supplemental Table S1).

Although many of the viral mRNAs lacked an A at position –1, nearly all (99.1%) had a G at position 0 (Figure 1B), and the remaining 0.9% that lacked this G were not considered further. For each read, the constant viral mRNA sequence, starting with the G at position 0, was trimmed from the 3′ end to leave the heterogeneous sequences, and any Gs that might have corresponded to untemplated Cs added by reverse transcriptase were trimmed from the 5′ end. The length distribution of the heterogeneous sequences matched the ∼10–13 nucleotide distribution observed previously in low-throughput analyses (Figure 1C) (7,9,25), suggesting that our method accurately captured influenza mRNA 5′ ends. Nevertheless, our heterogeneous sequences did not necessarily retain the ends of the cellular fragments that were snatched. At the 5′ end, leaders derived from host transcripts beginning with one or more G would have lost those Gs during our trimming step designed to remove untemplated nucleotides arising from the terminal cytosine transferase activity of reverse transcriptase. At the 3′ end, capped cellular fragments ending with a G that was used to prime transcription at the penultimate template nucleotide would have lost that G during our trimming step designed to remove the viral mRNA sequence.

In our initial analysis of the heterogeneous-sequence length distributions, the mode for HA, NS1, NA and NP (12 nt) was one nucleotide greater than that for MP, PA, PB1 and PB2 (11 nt). An analogous observation for the H3N2 virus is interpreted as evidence for different sets of host mRNAs contributing leaders for the different viral mRNAs (41).

Some heterogeneous sequences were represented by many more reads than others. These differences in read counts were moderately reproducible when comparing the biological replicates (Figure 1D, Spearman *r:* 0.68). Although we did not achieve the reproducibility typically observed for RNA-seq, which has the advantage of aggregating diverse reads over the length of each transcript, our method provided reasonably quantitative profiling of influenza heterogeneous sequences.

### The 3′ vRNA template influences the prime-and-realign register and frequency

**Figure 2.**
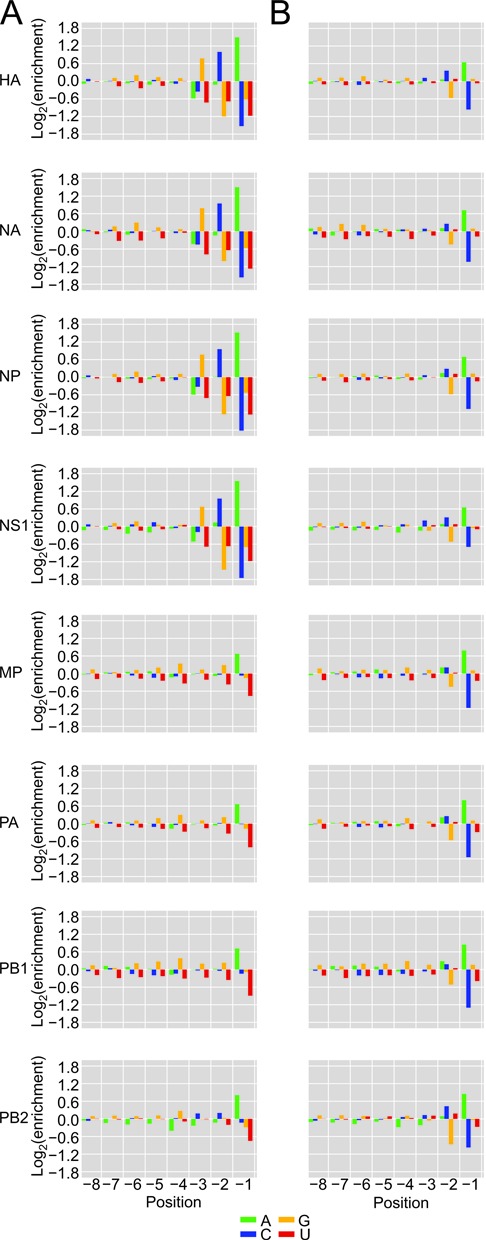
Nucleotide distributions at the 3′ ends of heterogeneous sequences and trimmed host leaders. (**A**) Nucleotide frequencies at the last eight positions of heterogeneous sequences before trimming residues attributed to prime-and-realign. At each position, enrichment was normalized to the overall nucleotide frequencies within 51-nt windows centered on all Gencode 17 TSSs. (**B**) Nucleotide frequencies after trimming residues attributed to prime-and-realign; otherwise, as in (A).

**Figure 3.**
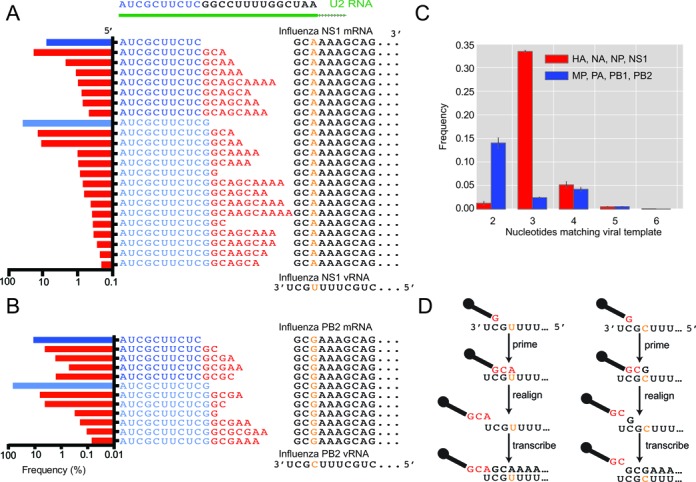
The potential contribution of prime-and-realign to the heterogeneous sequences. (**A**) A class of sequences most frequently prepended to NS1 mRNAs. These sequences begin with one of two related fragments (two shades of blue residues) both matching the 5′ end of U2. The U2-derived leaders are extended variable numbers of residues matching the vRNA (red residues), at frequencies indicated in the histograms at the left (blue bars indicating the fraction without any added nucleotides, red bars indicating the fraction of each species with added nucleotides). The sequences then continue with a contiguous match to the vRNA template (black residues, with the orange nucleotide indicating the SNP at position +2). (**B**) A class of sequences most frequently prepended to PB2 mRNAs; otherwise, as in (**A**). (**C**) Fraction of mRNAs with the indicated number of inserted nucleotides matching the vRNA. Frequencies from HA, NA, NP and NS1, which have a +2 U in the vRNA, are averaged, as are those from MP, PA, PB1 and PB2, which have a +2 C (red and blue, respectively); error bars, SD. (**D**) A prime-and-realign model that attributes the different numbers of inserted nucleotides to the influence of the +2 SNP in the vRNA.

Meta-analysis of heterogeneous-sequence nucleotide composition revealed two distinct signatures. A GCA trinucleotide was strongly enriched at the 3′ termini of the HA, NA, NP and NS1 heterogeneous sequences, whereas a single A was strongly enriched at the 3′ termini of the MP, PA, PB1 and PB2 heterogeneous sequences (Figure 2). Examination of the most abundant sequences revealed that this bias resulted in part from variable numbers of nucleotides appended to a common host sequence, as illustrated for the NS1 and PB2 mRNAs (Figure 3A and B, red nucleotides). These additional nucleotides perfectly reiterated short segments complementary to the 3′ ends of the viral RNAs, as expected for the prime-and-realign phenomenon (28–29,32,41). In theory, this pattern could also result from reiterative cap snatching, in which the virus snatched leaders from its own mRNAs, with the last event sometimes capturing a longer fragment than the initial one. However, the influenza polymerase avoids targeting its own mRNAs (53), and we found that cells infected for a longer time did not have a higher fraction of viral mRNAs with additional nucleotides (data not shown). With this evidence against reiterative cap snatching, we attribute these added nucleotides to the prime-and-realign phenomenon.

For the NS1 example, GCA was most commonly appended, which was followed by GCAA, GCAAA and others in decreasing frequency (Figure 3A). The frequent addition of GCA helped explain the enrichment for GCA at the 3′ termini of NS1 heterogeneous sequences (Figure 2A). Longer appended sequences that were consistent with two rounds of prime-and-realign, such as GCAGCAAAA, were also observed but tended to be less frequent (Figure 3A). Importantly, when realignment occurred on the NS1 template, the last nucleotide of the extended fragment was typically an adenosine, consistent with realignment guided by base pairing to the 3′ uridine of the vRNA, thereby supporting a model in which a single base pair between the leader and the template can prime transcription.

The prime-and-realign pattern differed for the PB2 mRNA (Figure 3B), which represented the mRNAs with a single A enriched at the 3′ termini of heterogeneous sequences (Figure 2). These mRNAs also tended to exhibit lower frequencies of prime-and-realign overall (Figure 3A–C). For the PB2 example, the most common extensions to the host sequences was GC instead of GCA, and less frequently, GCGA (Figure 3B).

To systematically study 3′ extensions of host leaders, we developed and implemented a computational algorithm to determine which heterogeneous sequences were extensions of other heterogeneous sequences in the dataset (Supplemental Methods). Although the algorithm was not informed of the viral genomic sequence, patterns consistent with one or two rounds of prime-and-realign were observed transcriptome-wide, suggesting prime-and-realign is not restricted to particular host leaders (Supplemental Figure S1). Furthermore, this algorithm recapitulated the observation that GCA 3′ extensions were most common on host leaders of HA, NA, NP and NS1 mRNAs, whereas GC was the most common extension on the host leaders of MP, PA, PB1 and PB2 mRNAs.

The different prime-and-realign frequency and patterns observed for the two sets of mRNAs perfectly correlated with a single-nucleotide polymorphism near the 3′ termini of their templates: 3′-UCGCUUU…-5′ for MP, PA, PB1 and PB2, instead of 3′-UCGUUUU…-5′ for HA, NA, NP and NS1 (54). Accordingly, we suggest that this template polymorphism imparts a major influence on the efficiency and outcome of the prime-and-realign mechanism. For example, following a 3-nt extension of a host fragment initially paired to only the 3′ uridine of the vRNA, a Watson–Crick pair between the terminal A of the host leader and the terminal U of the vRNA would mediate realignment on the NS1 template, producing a GCA extension, whereas a Watson–Crick pair between the terminal G of the host fragment and the penultimate cytosine of the vRNA would mediate realignment on the PB2 template, producing a GC extension (Figure 3D). Moreover, the dissociation of the nascent transcript required during realignment is expected to be less thermodynamically favored following extension with GCG compared to extension with GCA, which might explain the lower frequency of prime-and-realign for the MP, PA, PB1 and PB2 mRNAs. In summary, our results provided abundant evidence for the occurrence of prime-and-realign during influenza transcription initiation and revealed the influence of the viral template on the number of nucleotides added as well as the fraction of mRNAs affected.

### Similar host leaders are prepended to different viral mRNAs

**Figure 4.**
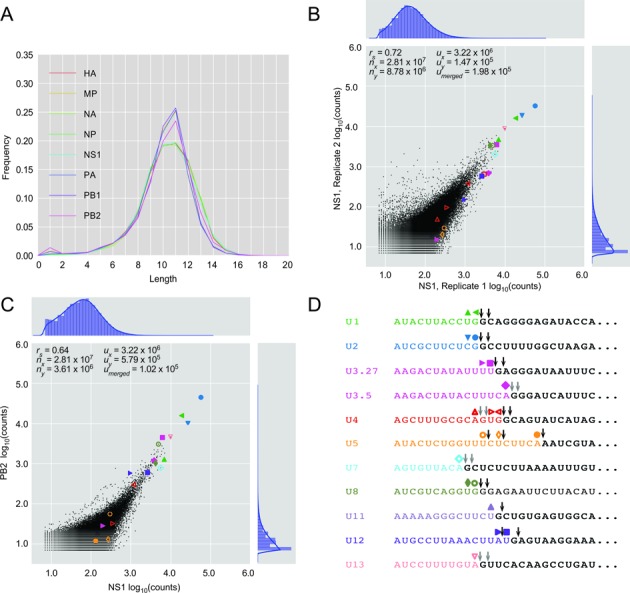
Different influenza mRNAs are prepended with similar sets of sequences that include leaders from snRNAs and snoRNAs. (**A**) Length distributions of host leaders after trimming nucleotides attributed to prime-and-realign. (**B**) Number of reads corresponding to the same host leader sequences in two biological replicates of NS1, 4 h.p.i., after trimming nucleotides attributed to prime-and-realign. Shapes indicate values for leaders mapping to the annotated 5′ ends of snRNAs and snoRNAs, colored as in panel (D). Otherwise, as in Figure 1D. (**C**) Number of reads corresponding to the same host leader sequences from NS1 and PB2 mRNAs, 4 h.p.i., after trimming prime-and-realigned nucleotides. Otherwise, as in (B). (**D**) Abundant host leaders corresponding to the annotated 5′ ends of snRNAs and abundant snoRNAs. The last nucleotide of each abundant trimmed host leader highlighted in (B) and (**C**) is indicated (colored shape), as is the presumed cleavage site for each of these host leaders (black arrows showing unambiguous sites or two gray arrows showing alternative cleavage sites for the same host leader).

Having identified the nucleotides inserted through the prime-and-realign mechanism, we trimmed these nucleotides from the 3′ termini of heterogeneous sequences to generate what we call ‘trimmed host leaders,’ which are more informative than untrimmed heterogeneous sequences when considering topics related to the early steps of cap snatching, such as the identities of the host transcripts, cleavage of the host transcript and initial priming. The nucleotide composition of trimmed host leaders converged on a common distribution with a slight enrichment of a cytosine at position –2 preceding a stronger enrichment for an adenosine at position –1 (Figure 2B). This pattern generalized the CA preference noted in a previous examination of several defined substrates (27). The convergence onto this pattern for all eight viral mRNAs also showed that differences in prime-and-realigned nucleotides explained essentially all the differences observed at the 3′ termini of heterogeneous sequences (Figure 2A), which indicates that the identity of the vRNA template does not influence host-fragment choice or utilization. The length distributions of the trimmed host leaders maintained a variance resembling that of the initial distributions (Figures 1C and 4A), indicating that prime-and-realign events were not a large contributor to the heterogeneity of host-leader lengths and that sequence preferences of the endonuclease itself causes leaders from some mRNAs to be of different lengths than those from others, as previously suggested (26). Nevertheless, accounting for prime-and-realigned nucleotides made the length distributions for trimmed host leaders for different mRNA much more similar to each other, with a uniform mode at 11 nt (Figure 4A), further indicating that vRNA identity does not affect PA-cleavage specificity or host-fragment utilization. Thus, accounting for the nucleotides inserted through prime-and-realign essentially erased the differences initially observed between viral mRNAs with respect both to the nucleotide composition at the ends of heterogeneous sequences and the length distributions of heterogeneous sequences, thereby providing a simple, alternative explanation for these two observations recently used to argue in favor of the idea that different sets of cellular transcripts contribute leaders to the different viral mRNAs (41).

When comparing the abundances of the trimmed host leaders in the two highest-complexity libraries, which were from the NS1 and PB2 mRNAs, the correlation approached that of biological replicates (Figure 4, Spearman *r* = 0.64 and 0.72, respectively), which further indicated that different viral mRNAs have caps-snatched from indistinguishable pools of cellular mRNAs. The recent study that concluded otherwise uses hierarchical clustering to suggest that each viral mRNA snatches caps from a specific subset of cellular mRNAs (41). To resolve this discrepancy, we performed pairwise comparisons between the dataset of each influenza mRNA and our highest-complexity dataset, that of NS1, and in parallel reanalyzed the data from the other study. All of the datasets from the other study, and indeed some ours, had striking bimodality in abundances of host leaders prepended to viral mRNAs (Supplemental Figures S2 and S3), which increased the intra-sample variance and might explain why clusters had been observed with hierarchical clustering. In our datasets, the bimodality was restricted to either early time-points during infection (30 and 45 min post-infection) or low-abundance influenza mRNAs, especially NA (Supplemental Figure S4). To investigate the source of the bimodality in our datasets, we compared the abundances of trimmed host leaders before and after collapsing PCR duplicates using UMIs. The bimodality was correlated with disproportionate amplification of a subset of high-abundance amplicons (Supplemental Figure S5). These results suggest that the variability in expression levels reported in the other study might also have been caused by a PCR artifact possibly related to low starting amounts of viral cDNA. The other study used additional enzymatic treatments to generate their libraries, which might have lowered their cDNA yield. Indeed we found that libraries had lower sequence complexity when using a CIP-TAP sequencing strategy similar to that used by Sikora *et al*. (Supplemental Methods, Supplemental Figure S6). Therefore, after considering nucleotides inserted through prime-and-realign and the distortions associated with low cDNA yield, we conclude that the different viral RNAs were generated using essentially the same set of host mRNAs.

### Influenza snatches caps from small nuclear RNAs and small nucleolar RNAs

Definitively assigning a trimmed host leader to a cellular gene proved challenging because mapping such a short sequence to the human genome typically resulted in thousands of hits. Therefore, we restricted the search space to 51-nt windows centered on the annotated transcription start sites (TSSs). To account for 5′-G trimming of the heterogeneous sequence, Gs were trimmed from the TSS 5′ ends as well. After also trimming potential prime-and-realigned nucleotides from the 3′ ends of heterogeneous sequences, the trimmed host leaders were mapped to these windows. Surprisingly, the most abundant leaders mapped precisely to the 5′ termini of snRNAs (Figure 4). Leaders corresponding to U1 and U2 were consistently the highest in abundance in all viral genes and at all time points measured (Supplemental Figure S3). To evaluate this result using an orthogonal method, we sequenced influenza 5′ ends using a variant of the recently described CIP-TAP strategy (41) (Supplemental Methods). Although the complexity of these libraries was lower than that of the template-switching libraries, leaders corresponding to U1 and U2 were still the most frequently sequenced (Supplemental Figure S6).

Trimmed host leaders mapping to all other well-annotated snRNAs and some snoRNAs were also present in high abundance, with the notable exception of those mapping to U6 (Figure 4D). Unlike the other sn/snoRNAs, which are transcribed by Pol II and whose nascent transcripts undergo the same capping as that of mRNAs, U6 is transcribed by Pol III and obtains a γ-methyl triphosphate, rather than an m^7^G, at its 5′ end (55). The absence of trimmed host leaders mapping to U6 was therefore consistent with both the known association of the viral RdRP with cellular Pol II and the strong preference for an m^7^G cap for cap-snatching (7,56–57). The abundance of the trimmed host leader corresponding to each of these small RNAs roughly correlated with the reported abundance of the mature sn/snoRNAs (58) (Supplemental Figure S7).

### A guanosine is often present immediately downstream of the trimmed host leaders

Although an A was generally enriched at the 3′ termini of the trimmed host leaders (Figure 2B), it was observed for only a few of the trimmed host leaders derived from abundant sn/snoRNAs. The most notable trend was instead observed at the position immediately downstream of the trimmed leader, which for all but one of these sn/snoRNAs was a G (Figure 4D). When considering that our annotation protocol would have trimmed away any 3′-terminal G present in the actual host fragment, this observation was consistent with the report that PA prefers to cleave after G (26). Leaders corresponding to U5, which lacks a G between nucleotides 9–24, ended with either an A at an unusual cleavage position, or an unusual nucleotide, U, at a more typical cleavage position, which suggested cleavage and usage of the U5 leader might be suboptimal (Figure 4D). Indeed, when considering the cellular abundance of the snRNAs (and disregarding the Pol III-transcribed U6), U5 was the least represented snRNA in the cap-snatching repertoire (Supplemental Figure S7).

The idea that a G was present at the 3′ termini of the most abundant host RNA fragments but was lost during our trimming procedure prompted a more general analysis of the nucleotide composition of host RNA substrates at the position immediately following trimmed host leaders. This analysis depended on accurate identification of the host RNA substrates, which was not possible for every leader. Although we trimmed the host leaders of any nucleotides that might not have originated from the host transcript and limited our mapping to regions of the genome proximal to annotated TSSs, the short length of these trimmed leaders inevitably led to some false-positive annotations of the host RNA substrates. Thus, before considering the nucleotide composition of the host RNA substrate downstream of the cleaved RNA fragment, we used more stringent thresholds to focus on the host RNA substrates identified with the highest confidence. To optimize these thresholds, we generated control sequences to assess the background of false-positive annotations associated with mapping very short sequences to a genomic index. These controls were trimmed host-leader sequences that had been shuffled randomly, except a G was not permitted at the first nucleotide (because our trimmed host leaders also lacked a G at this position), and the CG dinucleotide frequency was restricted to match its low frequency in the human genome. When applying increasingly stringent length thresholds for both the TSS window and the minimal length of the trimmed host leader, the signal-to-background ratio, as estimated by the number of matches to authentic host leaders compared to the number of matches to the controls, peaked at the annotated TSS (i.e. a TSS window size of 1) and at a minimum host-leader length of 10 nt (Supplemental Figure S8).

**Figure 5.**
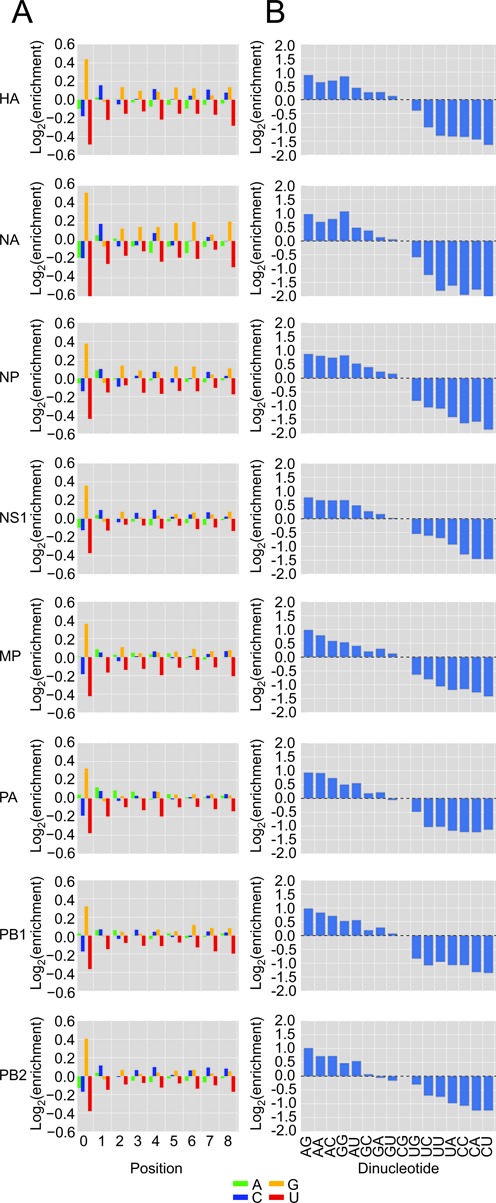
Inferred nucleotides near the cleavage sites of host transcripts. (**A**) Host transcript nucleotide composition immediately downstream of the trimmed host leaders. Analysis was limited to trimmed host leaders mapping precisely to Gencode 17 TSSs. The contribution of each downstream sequence was weighted in proportion to the rank of the corresponding host-leader abundance. Otherwise, as in Figure 2A. (**B**) Dinucleotide content at positions –1 and 0. Dinucleotide content statistics were collected from mapped host leaders from (A), using the same weighting as in (A). Enrichments were normalized to the dinucleotide composition of 51-nt windows centered on all Gencode 17 TSSs.

When focusing on only those host RNA substrates identified using these stringent thresholds, G was indeed enriched at position 0, i.e. the first position following the trimmed host leaders (Figure 5A). This enrichment was again consistent with the idea that many of the host RNA fragments differed from our annotated host leaders in containing a 3′-terminal G that primed across from the penultimate cytosine in the vRNA (26) but was trimmed away in our annotation procedure, which attributed a G at this position to the viral mRNA. To dissect the interdependencies of positions –1 and 0, which fall at or near the 3′-termini of the actual host RNA fragments, we examined the frequency of dinucleotides at these positions flanking the 3′ end of the trimmed host leaders (Figure 5B). As expected if the different viral RNAs were generated using essentially the same set of host mRNAs, these frequencies were highly correlated when comparing different viral mRNAs. The most enriched dinucleotides were AG, AA, AC, GG, AU, GC, GA, GU, CG and UG (underline indicating presumed 3′-terminal nucleotide of the actual host leader, acknowledging ambiguity for the dinucleotides ending in G; e.g. some of the AG dinucleotide presumably derived from cleavage after A and priming with a single pair to the terminal U of the vRNA template). These dinucleotides all had the possibility of a 3′-terminal G or A in the actual host leader, which have the potential to pair (as a Watson–Crick pair or G:U wobble) to either the penultimate C or the terminal U of the vRNA. Indeed, PA-catalyzed cleavage after the G of the most enriched dinucleotide, AG, would generate a host leader able to pair with both the 3′-terminal and penultimate vRNA nucleotides, which might strongly favour priming of transcription. This being said, the combined frequency of events attributed to a single base pair (AC, AA, AU, GC, GA, GU, UG and CG) was 1.9 times higher in aggregate than that of double base pair events (AG and GG), indicating that a single pair was often sufficient for priming. Most of these more enriched dinucleotides also had at least one G and thus the potential to derive from cleavage after G, as preferred by the PA endonuclease. The remaining six dinucleotides (UC, UU, UA, CC, CA, CU) lacked both features; i.e. they lacked the possibility of a 3′-terminal A or G in the actual host leader to facilitate priming, and they lacked the G favoured by the PA nuclease. Mis-mapped trimmed host leaders contributed part of the signal for each of the dinucleotides (which may explain the lower enrichment of UG and CG, compared to AC, AA, AU, GC, GA and GU). However, this mis-mapped background, estimated as ∼17% of the overall signal, could not explain all of the signal for these six that lacked both features, which indicates that some PA-catalyzed cleavage occurs after U and C, and that some of these host leaders ending in U or C are used to initiate productive transcription despite the mismatch to the template nucleotide (the 3′-terminal U of the vRNA), thereby generalizing our initial inferences made from the shorter leaders deriving from the U5 snRNA (Figure 4D).

## DISCUSSION

**Figure 6.**
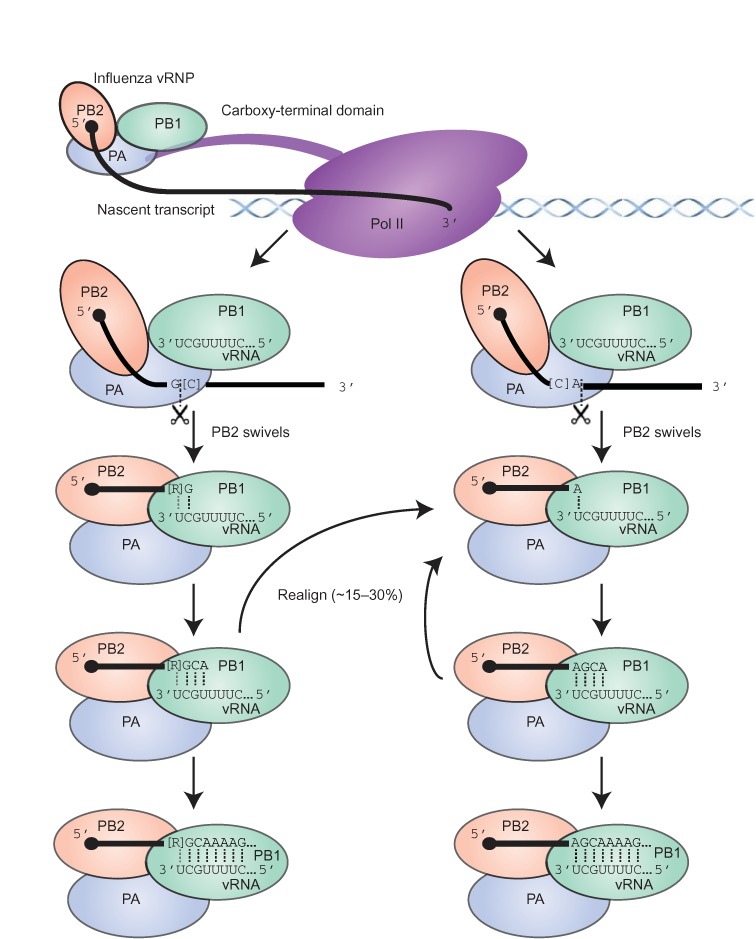
Summary model of influenza cap-snatching. The viral RNP binds to the CTD of Pol II (57) and PB2 captures a capped nascent transcript. Cleavage by the PA subunit occurs primarily after either a G (left pathway) or an A (right pathway), with possible preferences for flanking Cs (in brackets). Following cleavage, PB2 swivels to enable the 3′-terminus of the cleaved fragment to interact with the template in the PB1 subunit (62,63), allowing base pairing to the penultimate or last nucleotide of the vRNA template (left and right pathways, respectively), with potential pairing to both nucleotides if a purine (R in brackets) precedes a terminal G in the cleaved fragment (left pathway). Fragments ending in G can also pair to the terminal U of the template (not shown). One or more rounds of prime-and-realign can occur before processive transcription. Although fragments ending in U or C are sometimes used (not shown), they are not used as frequently as those ending in A or G, suggesting either that the inability of these fragments to pair with the relevant template residues might favour their dissociation prior to productive priming (not shown), or that the lack of G or A within the optimal window (∼10–13 nt from the 5′-terminus) might favor dissociation of the nascent transcript prior to cleavage (not shown). Dotted lines indicate base pairs; a gray dotted line indicates a potential base pair to the penultimate purine.

Our high-throughput sequencing and analysis of the heterogeneous sequences at the 5′ ends of influenza mRNAs support the model for influenza transcription initiation diagrammed in Figure 6. In the first step, PB2 binds the m^7^G cap of Pol II transcripts (11–13). Our unanticipated finding that many of the cap-snatched leaders derived from snRNAs/snoRNAs provided indirect evidence for cap snatching of nascent transcripts, presumably facilitated by the direct association between the viral polymerase and the C-terminal domain of Pol II (57). The alternative possibility, in which the virus snatches from mature transcripts, is disfavored because maturing spliceosomal snRNAs and some snoRNAs acquire a trimethylguanosine (m^3^G) cap, which binds to eIF4E with much less affinity than m^7^G (59) and thus would be less suitable for promoting viral mRNA translation. Moreover, cap snatching of nascent transcripts explains the presence of abundant fragments corresponding to U3, U8 and U13 (Figure 4C), despite localization of their mature forms to the nucleolus. It also explains the ∼3-fold greater abundance of leaders from U2 compared to U1, as U2 has the higher transcription rate (60) but accumulates to lower levels than U1 because of its shorter half-life (61) (Supplemental Figure S9). After accounting for nucleotides added through prime-and-realign, the lengths, nucleotide composition and identities of the host leaders were indistinguishable for the different viral mRNAs, which called into question the recently proposed influence of the viral template on the selection and utilization of the cellular transcripts (41). Therefore, we favor a simpler model in which the influenza polymerase initially recognizes nascent Pol II transcripts largely in proportion to their transcription rates, through interactions to Pol II and the co-transcriptionally added m^7^G cap (4), and with no influence of the viral template.

In the second step, the PA endonuclease cleaves the bound cellular transcript within a window approximately 10–13 nt from its 5′-terminus (Figure 4A, with the range adjusted for the frequent trimming of 3′- and 5′-terminal Gs from the actual host leader). Recent structural analyses of the influenza polymerase holoenzyme indicate that this cleavage occurs far from the 3′-terminus of the vRNA and far from the PB1 catalytic site, supporting the idea that cleavage preferences are directed by PA alone (62,63). Truncated PA endonuclease is reported to preferentially cleave after G *in vitro* (26). Our *in vivo* sequencing results were consistent with this observation, as the nucleotide immediately following the trimmed host leader was most commonly a G in the original host transcript (Figure 5). Our results further suggested that cleavage of the host mRNA also frequently occurs after A (Figure 2B), especially when it is preceded by C (27), which somewhat differed from the 4–9-fold preference for G over A observed *in vitro* (26). Whether this reflected a true difference between *in vitro* and the *in vivo* cleavage preferences is unclear, however, in part because our analyses were unable to deconvolute cleavage preferences from the subsequent preferences for leader utilization. For example, when examining the heterogeneous sequences ultimately found at the beginning of viral mRNAs, a strong preference for utilization of host leaders ending in A might have offset a strong preference for cleavage after a G. Alternatively, the difference might have reflected the limited number of substrate sequences tested *in vitro*, with the AU-rich RNA used with the truncated PA endonuclease lacking a CA cleavage site (26,27).

After PA cleaves the leader, PB2 swivels to place the 3′-terminus of the host leader into the PB1 polymerase catalytic site (62,63). Presumably leveraging a preference for cleavage after A or G, leaders ending in a purine preferentially prime viral transcription by virtue of their ability to form an A:U or G:U pair to the terminal U of the viral template or a G:C pair to the penultimate C of the template, with the leaders ending in AG priming particularly efficiently because of their potential to form two pairs with the template. This third step in the model is supported by two lines of evidence: (i) these priming positions were the two positions for which we observed the greatest nucleotide preferences, and these preferences were for A and G (Figures 2B and 5), and (ii) the most frequently realigned nascent viral transcripts all had a single base pair between their terminal residue and the viral genome (Figure 3)—if the initial priming and the subsequent realignment step are mechanistically equivalent, analogous base pairing would be involved in the initial priming step. Thus, we generalize insights made from earlier molecular and biochemical experiments that suggested a role for base pairing between the host leader and influenza vRNA 3′-terminus (28–30) by showing that this phenomenon systematically occurs on a transcriptome-wide scale. Moreover, we refine this insight, with our results indicating that a single base pair is usually used to prime, as has been proposed during cap-snatching by the tomato spotted wilt virus (37), also a negative-stranded segmented RNA virus but in a different family than that of influenza.

Despite the preference for creation/utilization of host fragments that end in A or G, we found evidence for some cleavage after U or C and utilization of these leaders that cannot form a Watson–Crick or G:U wobble pair to the relevant template residue (Figure 5B), as exemplified by the shorter U5-derived fragments (Figure 4D). The relative depletion of these U5 fragments (Supplemental Figure S7) and presumably fragments from other transcripts without a purine within the optimal cleavage window suggests that once initially bound, host transcripts are not all destined to contribute a leader to a viral mRNA; some transcripts are released, either before or after cleavage. With these off-pathway events, the sequence preferences for cleavage and priming presumably alter the cap-snatching repertoire, causing it to deviate from a strict correlation with Pol II transcription rate. Other deviations might occur as a result of Pol II pausing or other phenomena that might increase or decrease local concentration or initial accessibility of the cap.

After priming, some nascent viral transcripts dissociate from the template but are not released from PB2 and thus can reassociate in a different register, with a single-nucleotide polymorphism in the template influencing the efficiency and outcome of this phenomenon (Figure 3). Accounting for the resultant inserted nucleotides enables more accurate assessment of the previous steps of transcription initiation.

It might be tempting to speculate that cap snatching of snRNAs, which are ubiquitously expressed and evolutionarily conserved, might contribute to influenza's broad tissue and host tropism. Influenza might have even evolved cap-snatching with cleavage at an optimum distance from the cap centering on 11–12 nucleotides in order to preferentially access the Gs at these positions in U1 and U2. However, although these snRNAs contributed the most abundant leaders, their absolute abundance comprised <4% of the total sequence pool. Perhaps influenza is simply an opportunistic cap-snatcher. Its preference for cleavage after purine within a window of ∼4 nucleotides, with the subsequent priming using only a single base pair involving this terminal purine, is remarkably flexible and compatible with most Pol II transcripts.

Although the cap-snatching repertoire of H1N1 influenza has provided many insights, questions remain. Shuffled controls demonstrate uncertainty in identifying host leaders, even after setting stringent length thresholds (Supplemental Figure S8). Despite this uncertainty, the observation that the four most frequently sequenced leaders perfectly match snRNA/snoRNAs (Figure 4C) firmly establishes these noncoding RNAs as targets of influenza cap snatching. The uncertainty does, however, leave open the question of whether influenza might specifically target transcripts mediating the cellular response to infection. Putting aside the unanswered question of specific targeting, the observed global targeting, with a key preference for nascent transcripts, would broadly blunt any cellular transcriptional response to infection and in this way favor the virus without requiring additional specificity for particular targets. Our analyses are also unable to answer mechanistic questions, such as how PA directs preferential cleavage of the host mRNA and how the geometry of base pairing between the host leader and the vRNA is specified in the PB1 catalytic site. Nonetheless, our results provide an *in vivo* perspective to help inform subsequent structural and biochemical investigation of this process crucial for production of viral mRNA.

## SUPPLEMENTARY DATA

Supplementary Data are available at NAR Online.

SUPPLEMENTARY DATA

## ACCESSION NUMBERS

GSE65101.
