# Supplementary material for: Sequencing the cap-snatching repertoire of H1N1 influenza provides insight into the mechanism of viral transcription initiation
Source: Nucleic Acids Res. 2015 Apr 21;43(10):5052–64. doi: 10.1093/nar/gkv333 (PMC4446424; doi:10.1093/nar/gkv333)
Supplement: SUPPLEMENTARY DATA [file supp_43_10_5052__index.html]

Sequencing the cap-snatching repertoire of H1N1 influenza provides insight into the mechanism of viral transcription initiation — SUPPLEMENTARY DATA 

# Sequencing the cap-snatching repertoire of H1N1 influenza provides insight into the mechanism of viral transcription initiation

## SUPPLEMENTARY DATA

**Files in this Data Supplement:**

- SUPPLEMENTARY DATA
- SUPPLEMENTARY DATA
