# Supplementary material for: Sequencing the cap-snatching repertoire of H1N1 influenza provides insight into the mechanism of viral transcription initiation
Source: Nucleic Acids Res. 2015 Apr 21;43(10):5052–64. doi: 10.1093/nar/gkv333 (PMC4446424; doi:10.1093/nar/gkv333)
Supplement: SUPPLEMENTARY DATA [file supp_gkv333_nar-00165-r-2015-File008.pdf]

CIP-TAP Method

RNA was isolated from influenza-infected A549 cells as described in the Methods. 10 µg RNA was treated with Calf Intestinal Phosphatase (NEB) for 1 hour at 37°C in a 20 µl reaction containing 40 units Superase-In (Life Technologies), according to manufacturers' protocols. The reaction was then brought to 250 µl in 0.3 M NaCl, sequentially extracted with equal volumes of phenol:chloroform:isoamyl alcohol 25:24:1 pH 8 (Sigma Aldrich) and chloroform, and precipitated with ethanol and glycoblue (Life Technologies) overnight. The precipitate was pelleted, resuspended in water, then treated with Tobacco Acid Pyrophosphatase (Epicentre) according to manufacturer's protocol in a 10 µl reaction. RNA was extracted, precipitated, resuspended in an 8 µl mixture of 12.5% PEG 8000 (NEB), 10 µM 5′ adapter (Supplemental Table 2), 80 mM ATP, T4 RNA Ligase Buffer (NEB), 4 units Superase-In, and 8 units T4 RNA Ligase 1 (NEB), and incubated at room temperature overnight. The ligated product was boiled in 1x Gel Loading Buffer II (Life Technologies) and gel-purified on a thin denaturing 4% polyacrylamide gel, excising the slice corresponding to >750 nt but excluding the well. The eluate was precipitated, reverse transcribed, and amplified as described in the Methods section.

Oligonucleotides

Oligonucleotides were ordered from IDT and are summarized in Supplemental Table 2. Oligonucleotides with more than 60 bases were gel-purified prior to use.

Data Analysis

Preprocessing
Randomized nucleotides in the TSO and all subsequent contiguous Gs were used as unique molecular identifiers to collapse PCR duplicates (1). Cutadapt was used to trim sequences that perfectly matched the complement of positions 0–9 in the vRNA (2, 3) (NS1, HA, NP, NA: GCAAAAGCAG; MP, PA, PB1, PB2: GCGAAAGCAG). Reads without a perfect match and reads containing Ns within the remaining sequence were not considered, as were reads in which the remaining heterogeneous sequence was <9 or >15 nt. For Figure 1C, Figure 3A and B, and Figure 4A, the length restriction of <9 or >15 nt was not imposed. To account for prime-and-realign, any sequences with ≥2 3′-terminal nucleotides matching the 5′ end of the complement of positions 0–9 in the vRNA were iteratively trimmed up to four times from heterogeneous sequences. The resulting reads were designated trimmed host leaders. Trimmed host leaders < 9 nt were again discarded (except in Figure 4A). The number of read counts and nucleotides trimmed at the 5′ and 3′ end were retained in the FASTQ header as meta-information (4).

Mapping
Annotated TSSs were obtained from Gencode 17 (5) and iteratively trimmed at each 5′ terminus until the first nucleotide was not a G. A 51 nt window around each adjusted TSS was extracted using BEDTools and converted to a Bowtie index (6, 7). Cellular fragments were mapped to the custom Bowtie index, requiring a perfect, sense match (Bowtie parameters: -S –norc -p 24 –all –tryhard -v 0), converted to hg19 human genome coordinates in BAM format, and intersected with the Gencode annotations using BEDTools (6, 8, 9). If a read mapped to multiple paralogs, one paralog was chosen at random and the rest were removed from the dataset by collapsing on the Gencode 17 gene_name attribute. Reads mapping to multiple snRNA/snoRNA paralogs were also removed, accounting for somewhat variable gene_name attributes sometimes assigned to these paralogs.

To determine the background mapping of short sequences to TSS windows, we generated shuffled versions of trimmed host leaders, requiring that 1) the shuffled fragment does not start with a G, 2) the CG dinucleotide frequency is the same as the original sequence, and 3) the shuffled sequence is not the same as the original sequence. If all shuffled permutations of the original leader did not meet these criteria, the original leader was not considered.

Prime and realign

To efficiently find reads that are 3′ extensions of other reads in an unbiased manner, we developed an algorithm termed a "sequence tree." Consider a sequence *s* consisting of nucleotides *s*[0]…*s*[n] with associated read count c_s. Create a class consisting of nodes *i[u,c]* parametrized by associated nucleotide *u*, optional count *c*, and recursive child nodes *i*[A,c_A], *i*[C,c_C], i[G,c_G], and i[T,c_T] corresponding to the four possible nucleotides.

To insert a sequence into the tree, start at the base node. Find the child node *i* such that nucleotide *u* = s[0]. If it does not exist, create it. If the length of *s = 1*, set the count *c* of node *i* to c_s. Otherwise, recurse on this node using sequence *s*[1]…*s*[n]. Repeat for all sequences in dataset. To find the greatest common factor (GCF) nodes, examine the base node. For each child *i*, if it has an associated count *c*, it is a GCF. Otherwise, recurse on the children. To determine extensions of GCF nodes, find all nodes with counts beneath the GCF nodes. The program is available in the prime_and_realign.py file in the code repository.

Nucleotide composition

The nucleotide composition of cellular fragments with respect to their 3′ ends was determined by creating a position weight matrix (PWM) of nucleotide frequencies from the [–8, –1] interval and assessing the signal at each specific position and normalizing this signal to the overall nucleotide composition of the 51-nt Gencode 17 TSS windows. The interval [–8, –1] was chosen because positions [–15, –9] contain a high proportion of purines due to the nucleotide composition at Pol II TSSs and because some fragments are not that long.

The nucleotide composition downstream of the cellular fragments was inferred from mapping to annotated TSSs as described above and considering only those trimmed host leaders that mapped precisely to a TSS and had 10 or more nucleotides. Sequences were weighted in proportion to the ranked read count of the corresponding leaders and normalized to the overall nucleotide composition of TSS windows as before. Dinucleotide frequencies at positions –1 and 0 were inferred from the same set of mapped host leaders, imposing the same weighting but normalizing to the dinucleotide frequencies of the TSS windows rather than to the overall nucleotide composition.

Correlations

Trimmed host leaders with >6 reads in both of the two datasets being compared were considered. Correlations were visualized in tandem with histograms and kernel density estimates using default parameters from the seaborn package (http://stanford.edu/~mwaskom/software/seaborn/).

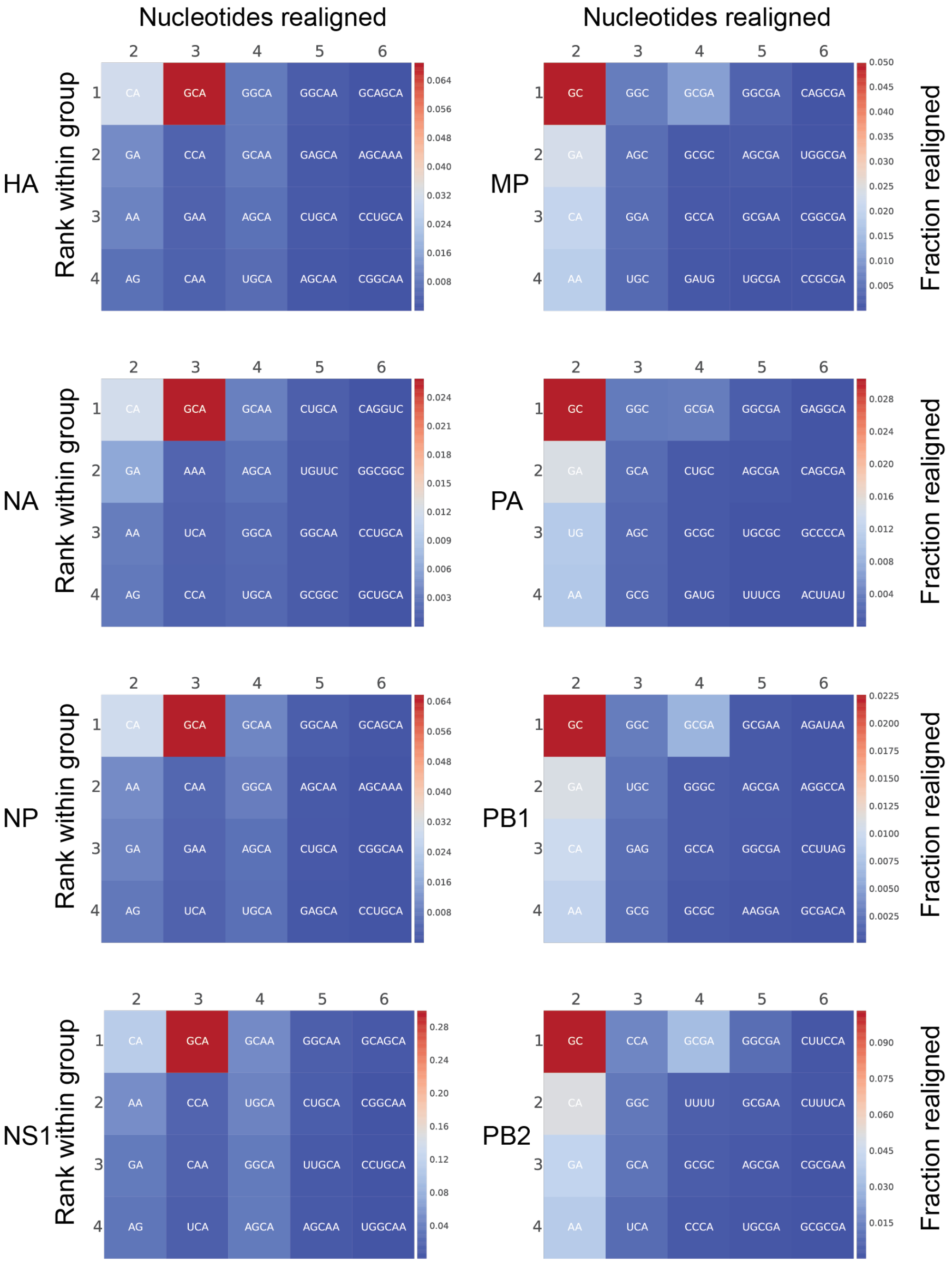

Supplemental Figure 1. Systematic analysis of nucleotides potentially added through the prime-and-realign mechanism. Heterogeneous sequences were inserted into a sequence tree (Supplemental Methods), and sequences that were 3′-extensions of “greatest common factor” sequences in the dataset were determined. Extensions were grouped by the number of nucleotides in the extension (columns) and sorted in decreasing order by frequency in which that extension was observed in the dataset (rows, colored according to the key).

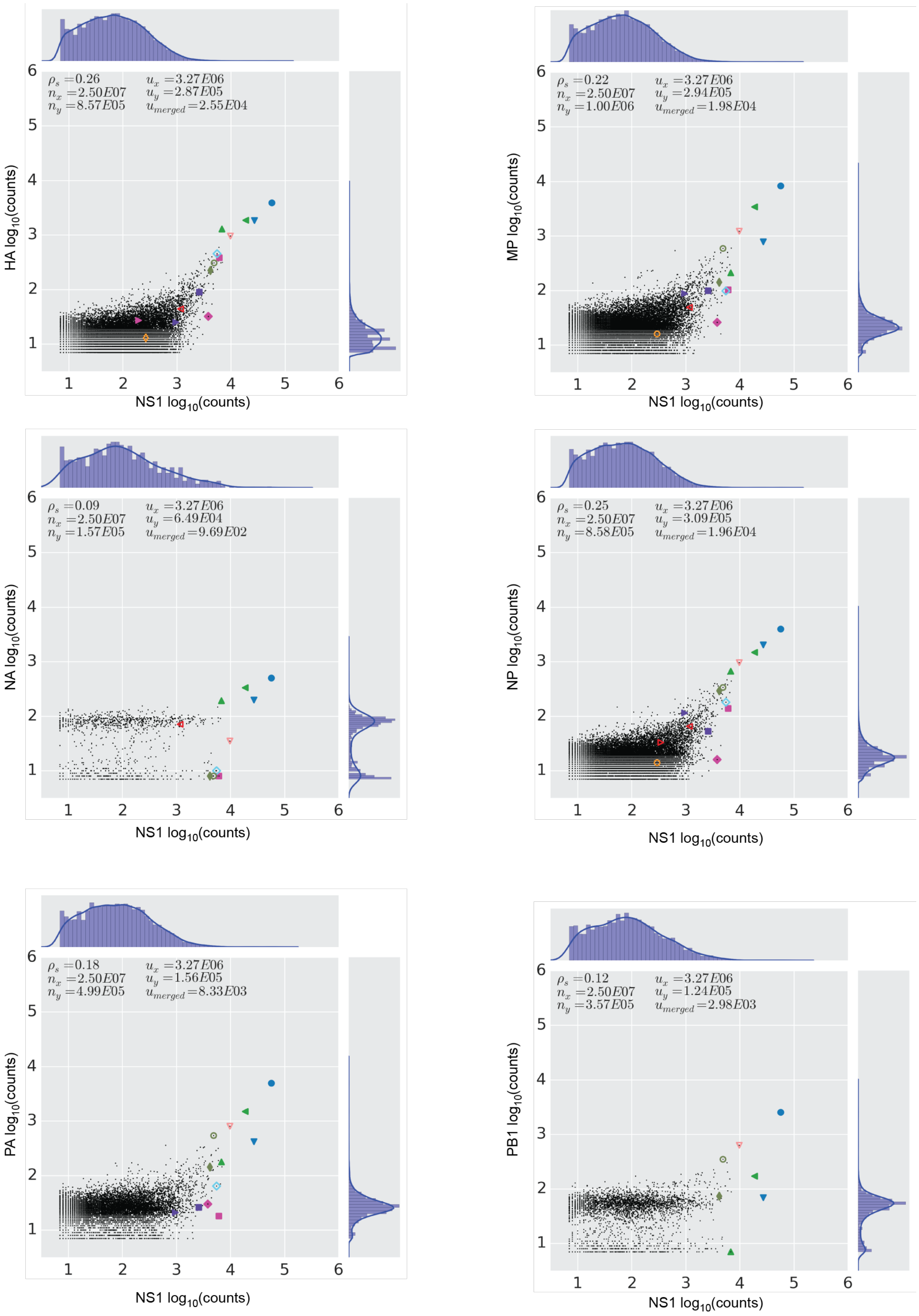

Supplemental Figure 2. Number of reads corresponding to the same host leaders from different influenza mRNAs, after trimming prime-and-realigned nucleotides, as in Figure 4C.

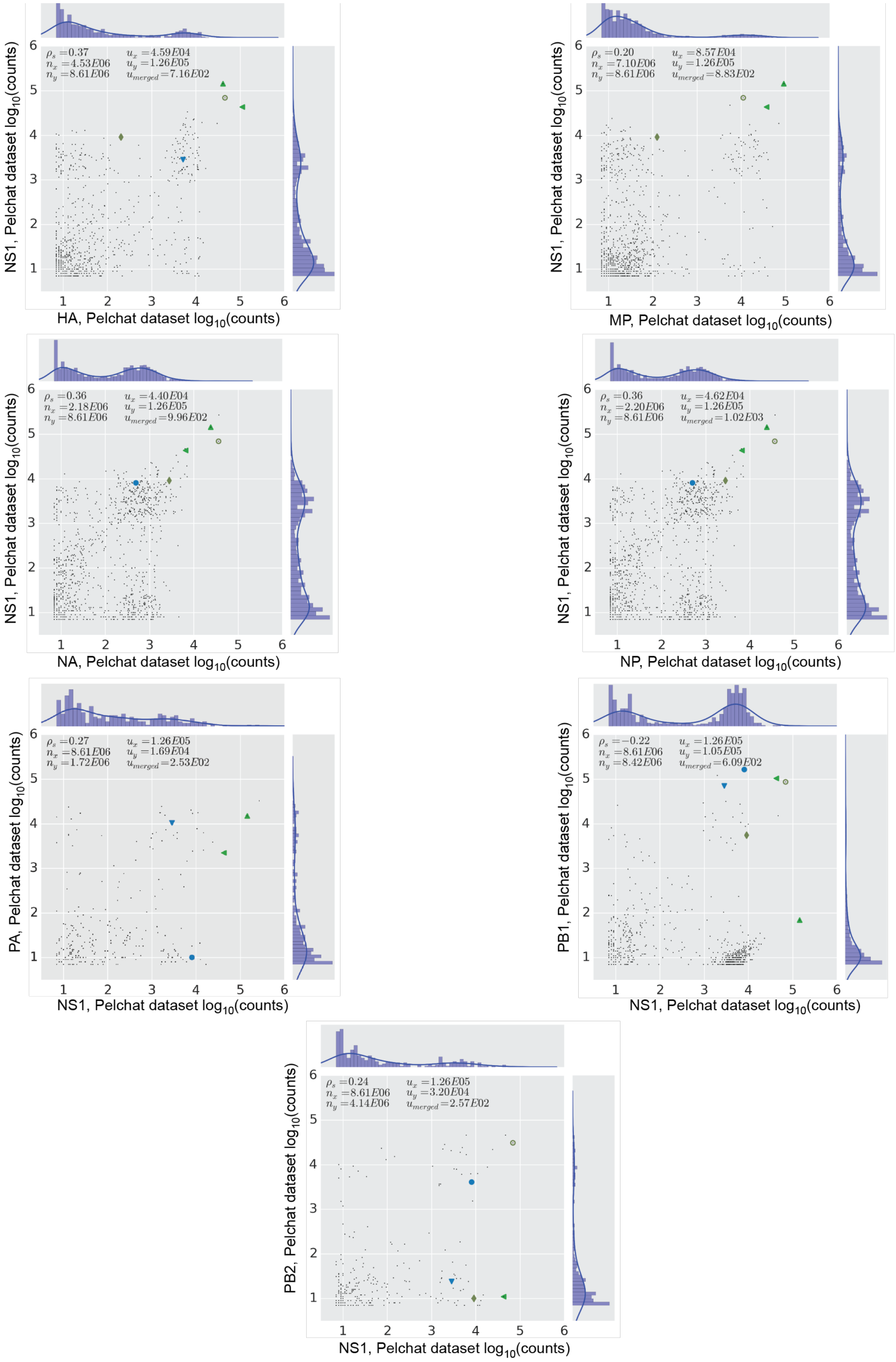

Supplemental Figure 3. Number of reads corresponding to the same host leaders from different influenza mRNAs, using the datasets from the Pelchat lab (10) and trimming prime-and-realigned nucleotides. Otherwise, as in Figure 4C.

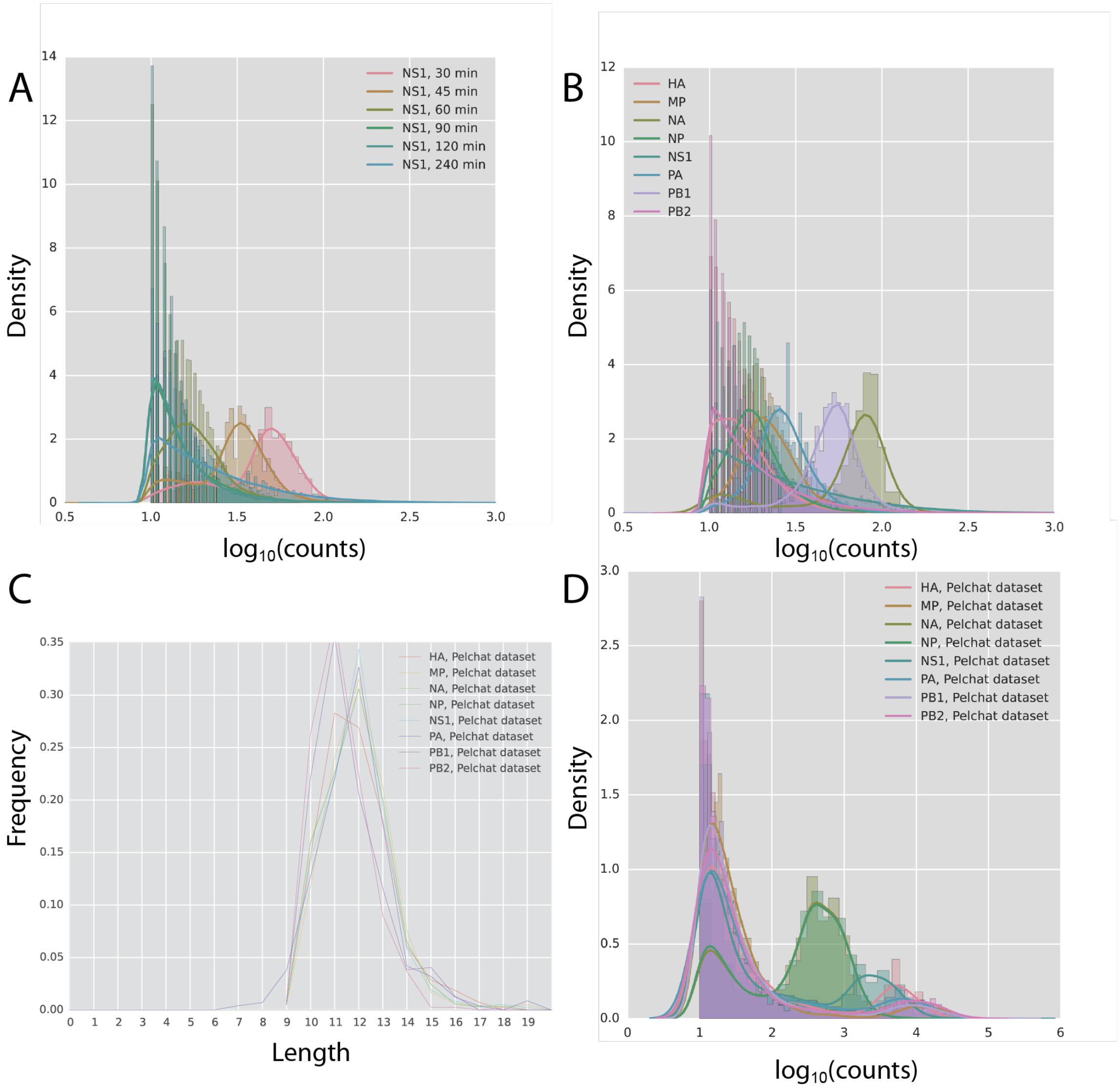

Supplemental Figure 4. Host leader abundances from different datasets. (**A**) Abundances of trimmed host leaders from NS1 mRNAs after the indicated time post infection. (**B**) Abundances of trimmed host leaders from each influenza mRNA 4 h.p.i. (**C**) Recapitulation of length distributions of datasets from Sikora et al. after reanalysis. (**D**) Abundances of trimmed host leaders from Pelchat lab datasets.

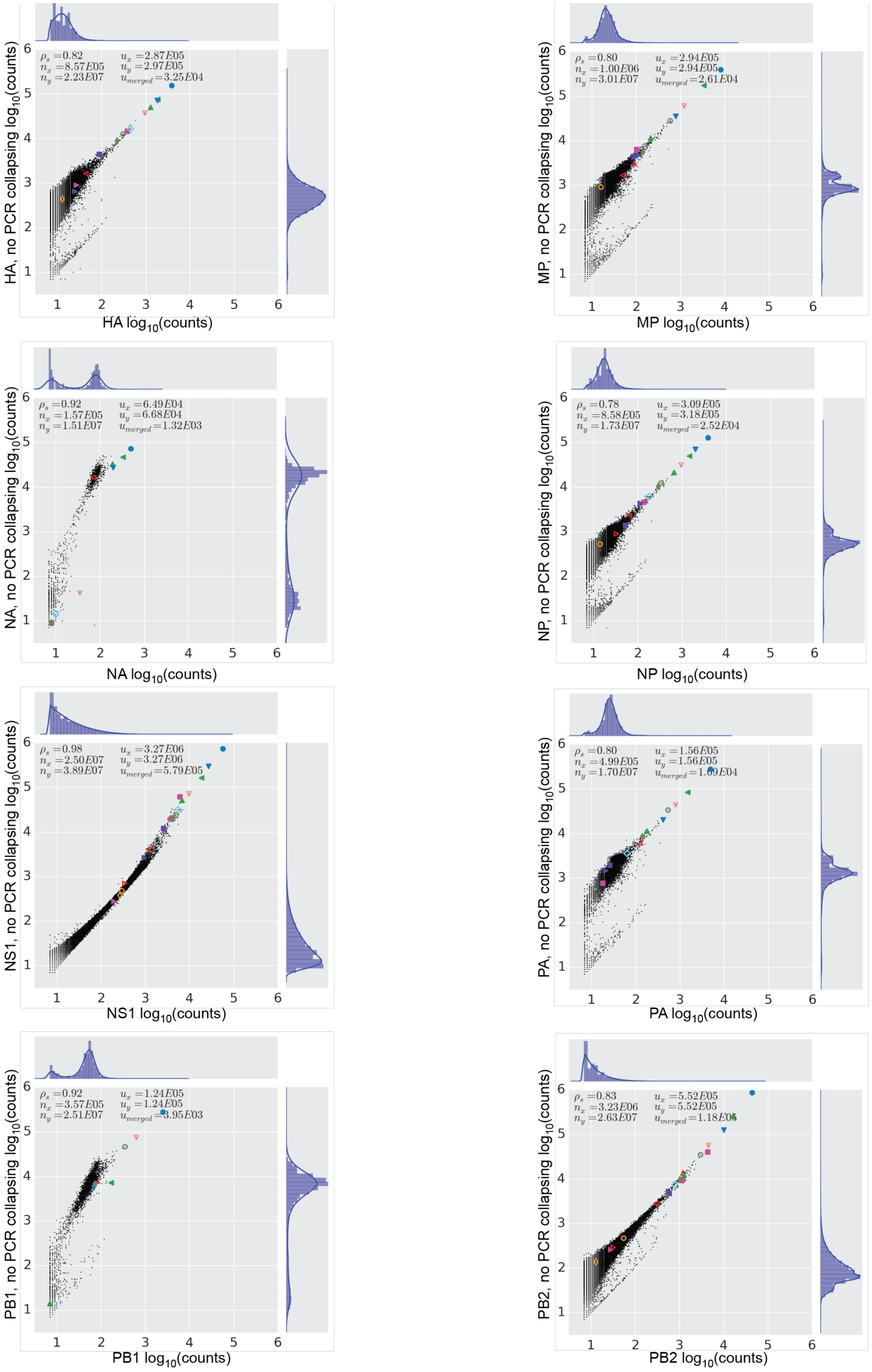

Supplemental Figure 5. Abundances of trimmed host leaders before and after barcode-enabled collapsing of PCR duplicates. Sequencing statistics and highlighting of snRNA sequences are as in Figure 4C.

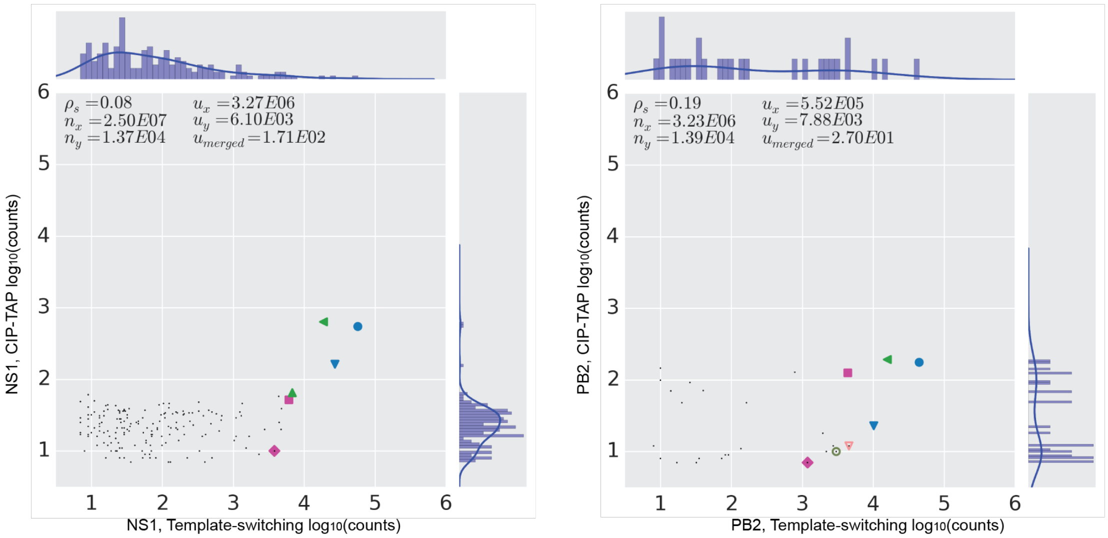

Supplemental Figure 6. Abundances of trimmed host leaders found using the CIP-TAP method compared to those found using the template-switching method. Sequencing statistics and highlighting of snRNA sequences are as in Figure 4C.

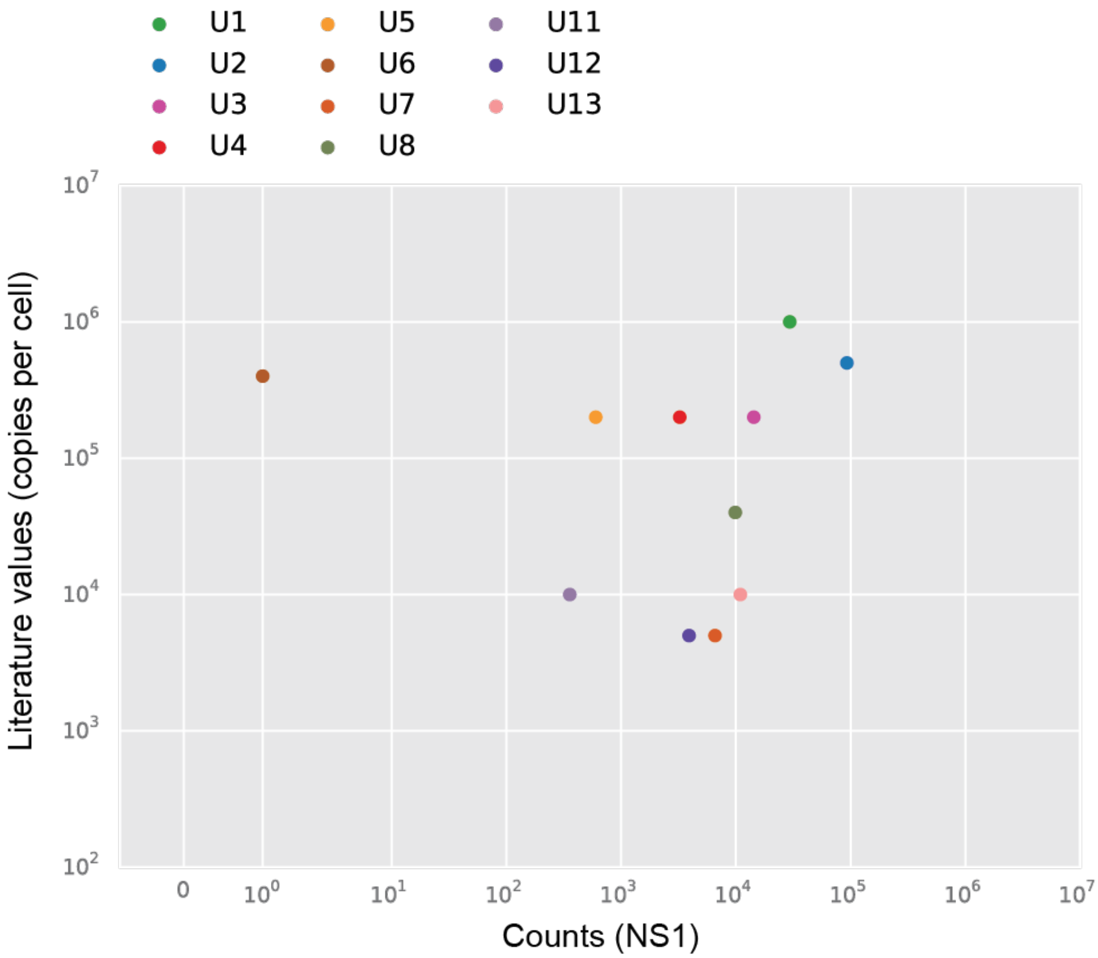

Supplemental Figure 7. Literature values of snRNA/snoRNA abundances (11) compared to abundances of host leaders corresponding to these snRNAs/snoRNAs in the NS1 template-switching dataset.

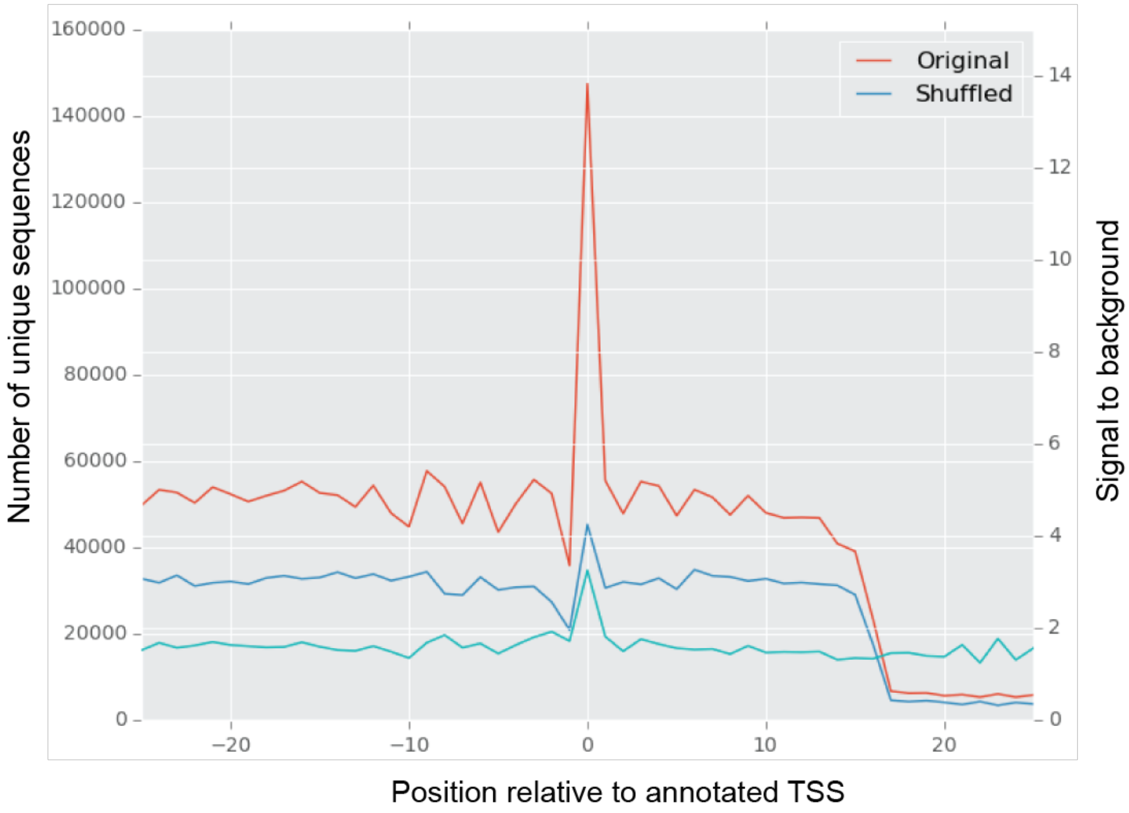

Supplemental Figure 8. Mapping trimmed host leaders to annotated TSSs, showing where actual host leaders (original) and control host leaders (shuffled) map relative to the adjusted annotated TSS. The increased signal for the in the controls at position 0 reflects the requirement that both the adjusted TSSs and the controls lack a 5′-terminal G. Sequences <10 nt were not considered. Using lower or higher cutoffs for sequence length reduced the signal-to-background ratio (calculated as original/shuffled).

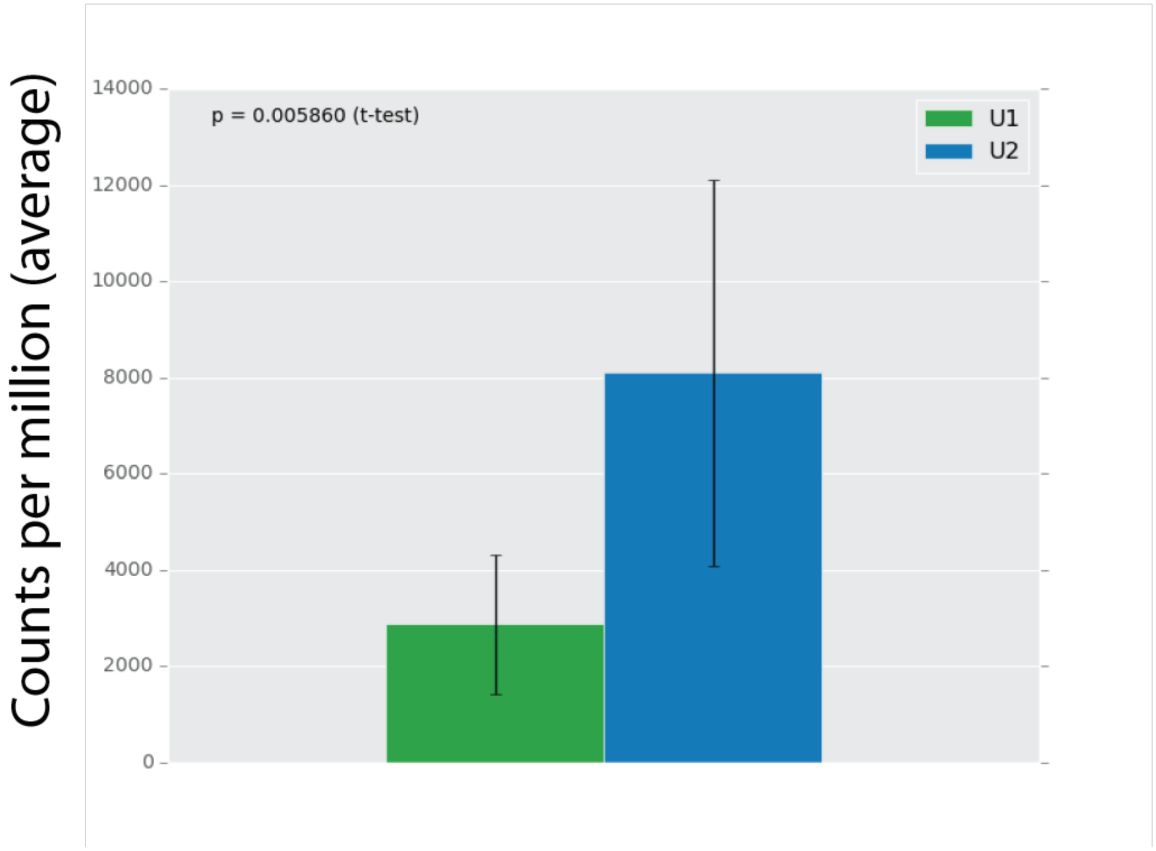

Supplemental Figure 9. Relative abundances of trimmed host leaders deriving from U1 and U2 across all of our template-switching datasets. Error bars, s.d.

References

1. Kivioja, T., Vähärautio, A., Karlsson, K., Bonke, M., Enge, M., Linnarsson, S. and Taipale, J. (2012) Counting absolute numbers of molecules using unique molecular identifiers. *Nat Methods*, **9**, 72–74.

2. Martin, M. (2011) Cutadapt removes adapter sequences from high-throughput sequencing reads. *EMBnet.journal*, **17**, pp. 10–12.

3. Plotch, S.J., Bouloy, M., Ulmanen, I. and Krug, R.M. (1981) A unique cap(m7GpppXm)-dependent influenza virion endonuclease cleaves capped RNAs to generate the primers that initiate viral RNA transcription. *Cell*, **23**, 847–858.

4. Cock, P.J.A., Fields, C.J., Goto, N., Heuer, M.L. and Rice, P.M. (2010) The Sanger FASTQ file format for sequences with quality scores, and the Solexa/Illumina FASTQ variants. *Nucleic Acids Res*, **38**, 1767–1771.

5. Harrow, J., Frankish, A., Gonzalez, J.M., Tapanari, E., Diekhans, M., Kokocinski, F., Aken, B.L., Barrell, D., Zadissa, A., Searle, S., et al. (2012) GENCODE: the reference human genome annotation for The ENCODE Project. *Genome Res.*, **22**, 1760–1774.

6. Quinlan, A.R. and Hall, I.M. (2010) BEDTools: a flexible suite of utilities for comparing genomic features. *Bioinformatics*, **26**, 841–842.

7. Langmead, B., Trapnell, C., Pop, M. and Salzberg, S.L. (2009) Ultrafast and memory-efficient alignment of short DNA sequences to the human genome. *Genome Biol.*, **10**, R25.

8. Li, H., Handsaker, B., Wysoker, A., Fennell, T., Ruan, J., Homer, N., Marth, G., Abecasis, G., Durbin, R.1000 Genome Project Data Processing Subgroup (2009) The Sequence Alignment/Map format and SAMtools. *Bioinformatics*, **25**, 2078–2079.

9. Lander, E.S., Linton, L.M., Birren, B., Nusbaum, C., Zody, M.C., Baldwin, J., Devon, K., Dewar, K., Doyle, M., FitzHugh, W., et al. (2001) Initial sequencing and analysis of the human genome. *Nature*, **409**, 860–921.

10. Sikora, D., Rocheleau, L., Brown, E.G. and Pelchat, M. (2014) Deep sequencing reveals the eight facets of the influenza A/HongKong/1/1968 (H3N2) virus cap-snatching process. *Sci Rep*, **4**, 6181.

11. Baserga, S.J. and Steitz, J.A. (1993) The Diverse World of Small Ribonucleoproteins. In Gesteland, R.F., Atkins, J.F. (eds), *The RNA World*. Cold Spring Harbor Monographs, Vol. 24.
